# Influence of Different Mixing Methods for Cementitious Capillary Crystalline Waterproofing Materials on the Self-Healing Capacity of Concrete Under Various Damage Types

**DOI:** 10.3390/ma18010159

**Published:** 2025-01-02

**Authors:** Haoyu Wang, Wei You, Guojin Ji, Liang Wang, Guoyou Yao

**Affiliations:** 1 Engineering Research Centre of Diagnosis Technology of Hydro-Construction, Chongqing Jiaotong University, Chongqing 400074, China; wanghaoyu0422@mails.cqjtu.edu.cn (H.W.); youwei@cqjtu.edu.cn (W.Y.); 2 Key Laboratory of Water Engineering Materials of Ministry of Water Resources, China Institute of Water Resources and Hydropower Research, Beijing 100048, China; 3 Powerchina Guiyang Engineering Corporation Limited, Guiyang 550081, China; liangwang_pcgecl@163.com; 4 Suzhou Guardex New Material Technology Co., Ltd., Suzhou 210500, China; yaoguoyou@rc-guardex.com

**Keywords:** Cementitious Capillary Crystalline Waterproofing Materials, concrete self-healing, multidimensional damage, crack self-healing

## Abstract

Cementitious Capillary Crystallization Waterproofing Material (CCCW), as an efficient self-healing agent, can effectively repair damage in concrete structures, thereby extending their service life. To address the various types of damage encountered in practical engineering applications, this study investigates the impact of different mixing methods for CCCW (including internal mixing, curing, and post-crack repair) on the multi-dimensional self-healing performance of concrete. The self-healing capacity of concrete was evaluated through water pressure damage self-healing tests, freeze–thaw damage self-healing tests, mechanical load damage self-healing tests, and crack damage self-healing tests. The results show that the curing-type CCCW mixing method exhibited the best self-healing effect in repairing water pressure, freeze–thaw, and load damages, with corresponding healing rates of 88.9%, 92.7%, and 90.5%, respectively. The internally mixed CCCW method was also effective for repairing load damage in concrete, while the repair-type CCCW mixing method demonstrated the weakest repair effect on these types of damage. For concrete with induced pre-existing cracks, the internally mixed CCCW method, after 28 days of water-immersion curing, exhibited a significantly higher crack self-healing ability, with a self-healing ratio of 333.8%. Optical microscopy observations revealed that the crack surfaces were almost fully sealed, with a substantial deposition of white crystalline material at the crack sites. Further analysis using scanning electron microscopy (SEM) and X-ray Diffraction (XRD) provided insights into the surface morphology and phase characteristics of the self-healed cracks, indicating that calcium carbonate (CaCO_3_) and calcium silicate hydrate (C-S-H) were the main products responsible for crack healing.

## 1. Introduction

Concrete is widely used as a construction material due to its high strength, good durability, and low cost [1,2]. However, the degradation of concrete structures poses a significant obstacle to their long-term service life [3,4], especially as internal or surface microcracks inevitably form under various complex factors such as temperature, shrinkage, and loading conditions. These microcracks [5,6,7] are difficult to accurately detect and identify. Without timely repair, external moisture and some harmful substances can penetrate into the interior of concrete structures through these cracks, potentially leading to internal steel corrosion [1,8], reduced concrete strength and durability, and shortened service life. Over time, larger cracks may develop, not only compromising the durability and safety of the concrete but also causing significant economic losses. Enhancing the durability of concrete is an engineering task with both economic and social benefits. Therefore, the timely and effective repair of these cracks is of paramount importance for the service life of concrete structures [9,10,11].

The self-healing process in concrete is generally classified into autogenous healing and autonomous healing mechanisms [12,13]. Autogenous healing primarily relies on the continued hydration of cementitious materials or the precipitation of minerals to repair cracks [14,15,16]. However, in ordinary concrete, the self-healing capacity is limited, leading to constraints in the crack width that can be effectively repaired, and the healing process can be prolonged. In contrast, autonomous healing involves the use of external healing agents that actively fill the cracks [17,18,19,20]. Numerous researchers have investigated the use of specific crystalline admixtures and mineral additives [18,21,22] to enhance or trigger the self-healing capability of concrete, significantly improving its ability to repair cracks.

Considerable research has been conducted on concrete crack self-healing and its related factors [17,18,23,24,25,26]. For example, reference [25] introduced different types of bacteria, discussed the different properties of bacterial concrete, and used it for crack self-healing. Additionally, reference [26] encapsulated bacteria in microcapsules for self-healing of concrete, evaluating its self-healing ability through crack-healing rate and water permeability. However, due to the limited lifespan of bacteria in concrete [27], challenges remain in the sustainable repair of concrete cracks and preventing capsule damage during casting [26]. Reference [18] studied the self-healing potential of cementitious materials, showing that additive specimens were favorable for calcium carbonate precipitation. In this regard, reference [21] analyzed the self-healing effect of crystalline admixtures in different environments on concrete, demonstrating that self-healing behavior depends on exposure conditions and the presence of crystalline concrete, proving water to be essential for healing reactions.

CCCW, usually prepared with active substances, are a type of self-healing material that can move along cracks and pores under the action of water, promoting the generation of crystalline products, achieving crack self-healing, and enhancing concrete performance [28,29,30]. Currently, many scholars have conducted extensive research on these materials. Reference [13] demonstrated that crystalline admixtures could reduce water permeability coefficients, decrease concrete surface resistivity, enhance chloride ion resistance, and accelerate crack width healing. Reference [31] studied the self-healing ability of CCCW mortar surface and internal cracks by changing crack width and water permeability tests, showing a significant decrease in relative permeability coefficient with added CCCW and good crack-healing ability. Researchers have also studied CCCW coatings, demonstrating their penetration depth in concrete and significant reduction in chloride ion diffusion coefficients, filling concrete voids and cracks, and improving structural density [32,33]. Furthermore, factors such as the type, dosage, and curing conditions of CCCW can significantly influence the self-healing performance of concrete cracks. For example, reference [34] examined the effect of different compositions of CCCW on the optimal dosage for concrete crack self-healing and determined that the optimal dosage is approximately 1%. Extensive research has shown that, under different exposure conditions, repair effects are best achieved with water-immersion curing [21,35] or wet–dry curing [36,37] conditions. Many researchers have also found that CCCW has a good repairing effect on cracks with widths less than 0.3 mm [38], with a complete closure of cracks observed. Extensive research on CCCW has demonstrated its excellent waterproof performance and shortened maintenance time due to its self-healing capabilities, without environmental concerns. In fact, many methods [18,31,37,39,40] have been proposed to evaluate the effect of CCCW on self-healing performance. Reference [41] pre-cracked cement-based composites with four different CCCW contents, followed by curing in water, Ca(OH)_2_ solution, and air. The transport properties of CCCW were evaluated through water absorption and rapid chloride ion penetration tests, while crack closure behavior was observed under a microscope. Additionally, reference [13] evaluated self-healing performance through parameters such as permeability, rapid chloride ion penetration rate, and resistivity. Reference [29] evaluated CCCW’s self-healing ability based on secondary permeability and pre-pressure self-healing rate, optimizing CCCW activation agent ratios.

In summary, while many researchers have demonstrated the excellent self-healing properties of CCCW in concrete, few studies have specifically examined the effect of CCCW on the self-healing performance of concrete structures with different types of damage. Concrete structures in hydraulic engineering often experience various types of damage throughout their service life, such as water pressure damage, freeze–thaw damage, mechanical load damage, and cracking, commonly observed in hydraulic tunnel linings. The incorporation of CCCW as a repair mechanism offers a promising maintenance approach for these structures. However, most researchers have primarily focused on using CCCW as an internal admixture. In addition to being used as an initial internal additive, CCCW can also be applied during the concrete hardening process or after damage occurs for repair purposes. Nevertheless, the effectiveness of these different application methods in addressing various types of damage has not yet been quantitatively compared. Therefore, a comprehensive evaluation of the self-healing performance of CCCW concrete, applied through different methods, is urgently needed for the common damage types encountered in hydraulic engineering concrete structures. This will enable the selection of the optimal CCCW application method based on the specific types of damage encountered in real-world engineering, ensuring the safe operation of concrete structures.

This study investigates the multi-dimensional self-healing performance of CCCW under common types of damage in concrete structures, including water pressure damage, load-induced damage, freeze–thaw damage, and cracking. Three different CCCW application methods are examined: internal mixing, external application, and post-damage surface coating. To quantitatively analyze the self-healing effects of different CCCW application methods on various types of concrete damage, this study proposes four self-healing capacity ratios: water pressure damage self-healing capacity ratio ($R_{w}$), freeze–thaw damage self-healing capacity ratio ($R_{f}$), load damage self-healing capacity ratio ($R_{p}$), and crack self-healing capacity ratio ($R_{c}$). For a direct observation of the crack-healing process, pre-cracked specimens were subjected to water-immersion curing, and changes in crack width before and after curing were observed under a microscope. The crack closure ratio (γ) was used to describe the self-healing performance of CCCW on cracks. Finally, XRD and SEM were employed to analyze the self-healing products and processes of CCCW, and a self-healing mechanism was proposed. The results of this study provide guidance for selecting appropriate CCCW application methods based on different types of concrete damage in practical engineering, helping to restore the performance of concrete structures and ensure their proper functioning.

## 2. Experimental Program

In this study, the symbols used and their corresponding meanings are shown in Table 1.

### 2.1. Materials

Concrete with a water to cement ratio of 0.55 and a sand ratio of 0.34 was made using ordinary Portland cement (PO 42.5, HONGSHI HOLDING GROUP, Jinhua, Zhejiang, China) and coarse aggregate. Different incorporation methods of CCCW (Suzhou Guardex New Material Technology Co., Ltd., Suzhou, China) were employed for internal mixing, curing, and repair. The CCCW used for internal mixing mainly consisted of inorganic nano-silicate with a small amount of hydration heat inhibition component, with a dosage of 2% of the cement mass, replacing an equivalent mass of water. The CCCW used for post-formwork curing and damage repair was the same material, mainly composed of inorganic nano-silicon ions, sprayed on the surface of the concrete specimens at a dosage of 250 mL/m^2^.

### 2.2. Mixture Proportions and Mixing Procedures

Table 2 lists the mix proportions of the concrete. PC0 represents the reference concrete, i.e., the control sample without CCCW. FI1, FC1, and FR1 represent specimens for internal mixing, curing, and repair, respectively, with the CCCW usage methods as specified in Section 2.1. For different damage types under standard curing, S1, S2, S3, and S4 represent water pressure damage, freeze–thaw damage, load damage, and cracking damage, respectively. The crack self-healing test also employed water-immersion curing, denoted as S5. Water pressure damage was applied using a permeability testing device, with the concrete specimen dimensions being 175 mm × 185 mm × 150 mm. Freeze–thaw damage was induced using a rapid freeze–thaw testing apparatus, with concrete specimens measuring 100 mm × 100 mm × 100 mm. Load-induced damage was applied using a compression testing machine, with the specimen dimensions being 100 mm × 100 mm × 100 mm. Cracking damage was also induced using the compression testing machine, with the concrete specimens being cylindrical with an inner diameter of 10 cm and a height of 5 cm.

**Figure 1:**
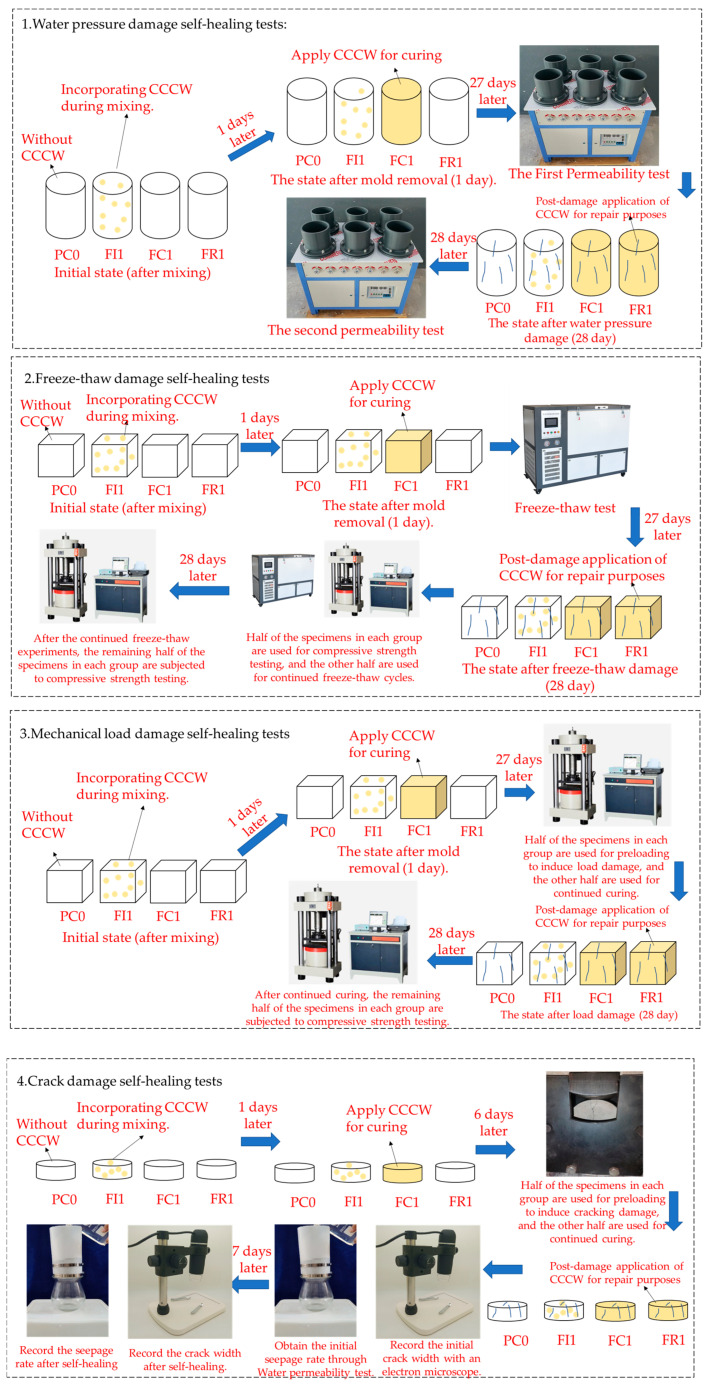
Schematic diagram of specimen preparation and experimental procedures.

The concrete casting and experimental testing were conducted in accordance with the standard test method for the self-healing performance of cement concrete (T/CECS 913-2021) [42] and Standard for Test Methods of Concrete Physical and Mechanical Properties (GB/T 50081-2019) [43], respectively. The CCCW for internal mixing should be added to the concrete simultaneously with water and water-reducing agents. The concrete was demolded 24 h after casting and then placed in a standard curing chamber with a controlled temperature of 20 ± 2 °C and 95% relative humidity (RH). CCCW for curing was sprayed onto the surface of the specimens after demolding for use. External repair CCCW was applied to the surface of the specimens after they underwent different types of damage. The specimen preparation and experimental procedures for CCCW on specimens with various types of concrete damage in this study are illustrated in Figure 1.

### 2.3. Permeability Test

**Figure 2:**
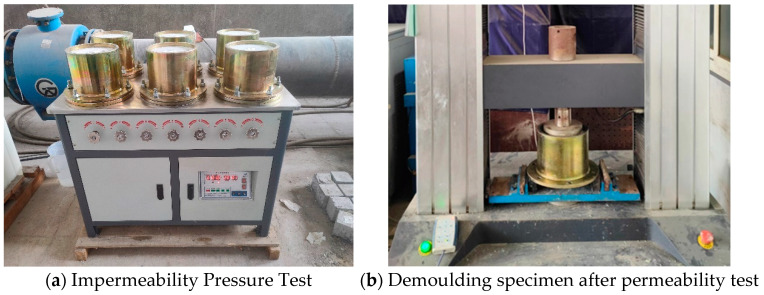
Schematic diagram of impermeability test.

According to the permeability test requirements specified in standard [43], each group contained six concrete permeability test specimens (175 mm × 185 mm × 150 mm) prepared according to the mix proportions. Before the first permeability test, the surface of the test specimens was dried, and the sides were sealed with wax and rosin. The specimens were then mounted on the concrete permeability tester (Schenck Weijie Instrument Equipment Co., Ltd., Beijing, China), as shown in Figure 2a. The valve of the permeability tester was opened, and the water pressure was increased to 0.1 MPa. The pressure was increased by 0.1 MPa every 8 h, and the water seepage at the top of the specimens was observed in real time. The pressure corresponding to the seepage was recorded. When all specimens exhibited seepage, the test was concluded, and the seepage pressure of the third specimen was reduced by 0.1 MPa, which was recorded as the first permeability pressure of the concrete (28 days). The tested specimens were then demolded and cured, as shown in Figure 2b. After curing, the second permeability pressure (56 days) was tested following the same procedure as the first test.

To characterize the effect of different CCCWs on the recovery of concrete permeability pressure, the permeability pressure was used as a comparative value to obtain the permeability pressure recovery rate, determined according to the following equation [42]:

(1) $$\lambda_{w}=\frac{P_{2}}{P_{1}}\times 100\%$$

where $\lambda_{w}$ is the permeability pressure recovery rate, and $P_{1}$ and $P_{2}$ are the first and second permeability pressures, respectively.

The self-healing performance of CCCW on concrete water pressure damage was characterized by the water pressure damage self-healing capability ratio [42]:

(2) $$R_{w}=\frac{\lambda_{ws}}{\lambda_{wc}}\times 100\%$$

where $\lambda_{ws}$ and $\lambda_{wc}$ are the permeability pressure recovery rates of CCCW-incorporated and reference concrete, respectively.

### 2.4. Freeze–Thaw Test

Concrete was cast into 100 mm cubic molds. After curing, six specimens from each group were selected for continued curing as control specimens, while the remaining six were placed in a concrete rapid freeze–thaw tester (Gangyuan Test Instrument Factory, Tianjin, China). After the specified freeze–thaw cycles, three corresponding freeze–thaw and control specimens were selected, and their compressive strength was tested according to [43]. The remaining three specimens were further cured and tested for compressive strength at the designated age. The notation 28-F100 denotes the compressive strength of specimens after 100 freeze–thaw cycles ($f_{c}$), 28-F100S denotes the compressive strength of control specimens at the same age ($f_{i}$), 28-F100-28 denotes the compressive strength of specimens after 100 freeze–thaw cycles and 28 days of curing ($f_{n}$), and 28-F100S-28 denotes the compressive strength of control specimens at the same age as the freeze–thaw cured specimens ($f_{0}$). The freeze–thaw damage compressive strength retention rate (η), the freeze–thaw damage compressive strength recovery rate ($\lambda_{f}$), and the freeze–thaw damage self-healing capability ratio ($R_{f}$) [42] are defined as

(3) $$\eta =\frac{f_{c}}{f_{i}}\times 100\%$$

(4) $$\lambda_{f}=\frac{f_{n}}{f_{0}}\times 100\%$$

(5) $$R_{f}=\frac{\lambda_{fs}}{\lambda_{fc}}\times 100\%$$

where $\lambda_{fs}$ and $\lambda_{fc}$ are the freeze–thaw damage compressive strength recovery rates of CCCW-incorporated and reference concrete, respectively.

### 2.5. Pre-Pressure Test

The pre-pressure test reflects the self-healing performance of CCCW on compressive strength recovery after pre-pressure. Each group had 12 specimens (100 mm cubic) cast, and, after 28 days of curing, 9 specimens were selected, with the remaining 3 kept for continued curing. Three of the nine selected specimens were tested for compressive strength. To induce microcracks without causing major cracks, 80% of the compressive strength load was applied to the remaining six specimens, maintained for 30 s, and then unloaded for further curing. After the designated age, the compressive strength of the load-damaged and control specimens was tested. The notation 28-P80-28 denotes the compressive strength of load-damaged specimens after 28 days of curing ($f_{l}$), and 28-P80S-28 denotes the compressive strength of control specimens at the same age ($f_{l0}$). The load damage compressive strength recovery rate ($\lambda_{p}$) and the load damage self-healing capability ratio ($R_{p}$) [42] are defined as

(6) $$\lambda_{p}=\frac{f_{l}}{f_{l0}}\times 100\%$$

(7) $$R_{p}=\frac{\lambda_{ps}}{\lambda_{pc}}\times 100\%$$

where $\lambda_{ps}$ and $\lambda_{pc}$ are the load damage compressive strength recovery rates of CCCW-incorporated and reference concrete, respectively.

### 2.6. Crack Creation and Healing

**Figure 3:**
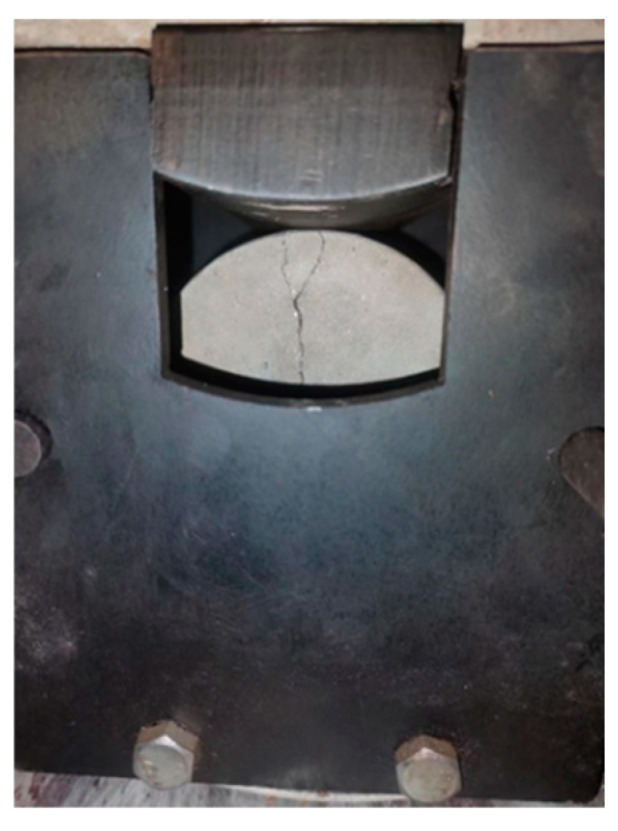
Illustration of the pre-cracked specimen.

Concrete specimens were prepared according to the mix proportions listed in Table 1 and poured into molds (cylindrical with an inner diameter of 10 cm and a height of 5 cm). Each group required eight specimens. The top surface was covered with plastic film. After 7 days of standard curing, the surface moisture was wiped off, and sealing tape was wrapped around the specimens to prevent detachment during loading. Pre-cracks were induced using a pressure testing machine (Wanxiang Instrument Equipment Co., Ltd., Hengshui, China) [41], and the process was stopped when cracks appeared. A schematic diagram of the crack fabrication for the specimens is shown in Figure 3. The pre-cracked specimens were rewrapped with sealing tape to prevent detachment at the cracks. To ensure similar initial cracks across groups, the seepage rate was used for control (see Section 2.6). The initial crack width was measured with a microscope, and samples meeting the seepage rate requirements were selected for crack width measurement at the same location.

### 2.7. Water Permeability Test

**Figure 4:**
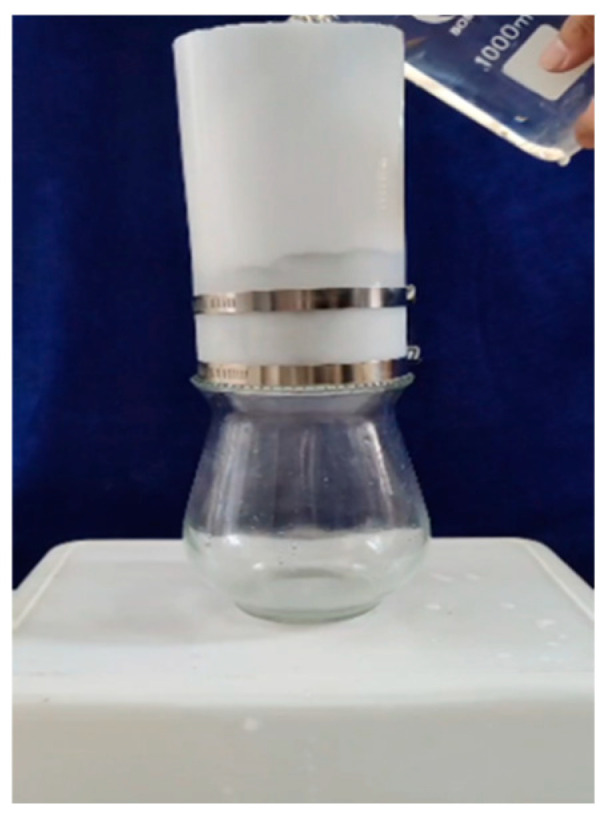
Illustration of the setup of the water permeability test.

The water permeability testing method and apparatus described in reference [31] are used in this study. Figure 4 illustrates the water permeability testing device employed in the current research. The top of the setup consists of a rubber cylinder with an inner diameter of 100 mm and a height of 200 mm. The bottom of the rubber cylinder is equipped with two steel hoops to ensure a tight fit with the pre-cracked specimen, preventing water leakage from the sides. The lower end of the setup is a water-collecting glass cup. An amount of 1000 mL of water was poured into the rubber cylinder from the top, and the time taken for all the water to seep out was recorded. The seepage rate K was calculated according to Equation (8) [42]. The initial seepage rate was measured before healing, with a minimum of 150 mL/min, and the initial seepage rate of each group of specimens and reference specimens should not exceed 10% of their average value, ensuring control over the initial seepage rate of the pre-cracked specimens. The permeability test was repeated after 7 or 28 days of healing.

(8) $$K=\frac{1000-V}{t}$$

where V is the remaining water volume in the rubber cylinder (mL), and t is the test duration (min).

To characterize the self-healing effect of different CCCWs on cracks using seepage rate, the initial seepage rate and final seepage rate were used as comparative values. The relative permeability coefficient (β) and crack self-healing capability ratio ($R_{c}$) [42] are suggested as follows:

(9) $$\beta =\frac{K_{n}}{K_{0}}\times 100\%$$

(10) $$R_{c}=\frac{100-\beta_{s}}{100-\beta_{c}}\times 100\%$$

where $K_{n}$ and $K_{0}$ are the final and initial seepage rates, respectively.$\beta_{s}$ and $\beta_{c}$ are the relative permeability coefficients of CCCW-incorporated and reference concrete, respectively.

### 2.8. Crack Width Measurements

Numerous scholars [21,23,35] have shown that the repair effect under water-immersion curing is better than standard curing, and, to observe the entire process of crack self-healing, the crack width of each sample at different curing ages was measured using an optical microscope. The crack location was marked with a marker to ensure consistent measurements, and photographs of the cracks were taken for visual comparison before and after healing. All measurements were conducted under water-immersion curing. For a given measured crack width, the crack closure rate (γ) [42] is defined as

(11) $$\gamma =\frac{W_{0}-W_{n}}{W_{0}}\times 100\%$$

where $W_{0}$ is the initial width of the pre-crack, and $W_{n}$ is the crack width after n days of self-healing.

### 2.9. Microscopic Observation

To determine the phase composition of the healing products, crystals from the crack area of water-immersed pre-cracked specimens were carefully scraped from both blank and CCCW samples, immersed in anhydrous ethanol to terminate hydration, dried in an oven at 60 °C for 24 h, and ground for X-ray Diffraction (XRD) testing. The samples were scanned from 5° to 90° using an X-ray Diffractometer Ultima IV manufactured by Rigaku, Tokyo, Japan.

The morphology of the healing products was observed using scanning electron microscopy (SEM). Small pieces were cut from the crack area of specimens cured for 28 days in water. The cut pieces were subjected to water-stop hydration and dried for 24 h. Before SEM testing, the samples were fixed, gold-sprayed, and then imaged using the Zeiss Gemini Sigma 300 VP SEM-type field emission scanning electron microscope produced by Carl Zeiss AG, Oberkochen, Germany.

## 3. Results and Discussion

### 3.1. Permeability Experiments

**Figure 5:**
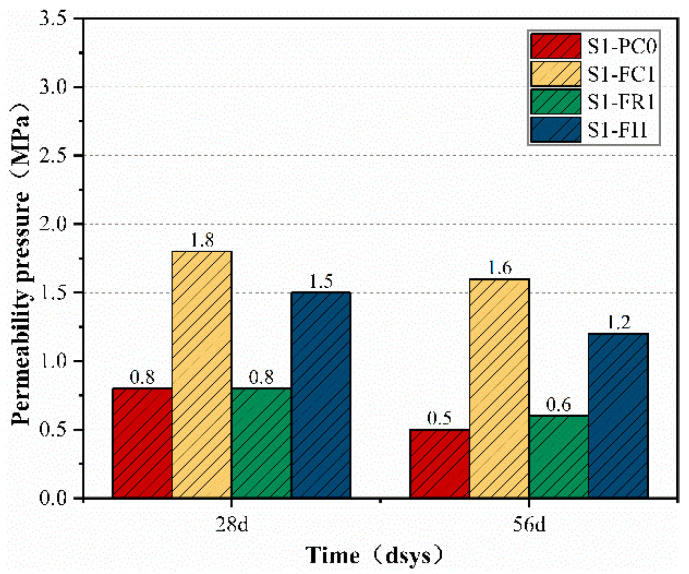
First (28 d) and second (56 d) permeability pressures.

The permeability pressures of the impermeable specimens at 28 d and 56 d under the same curing conditions were compared with those of the reference group (i.e., Figure 5). The results indicate that, regardless of the first or second permeability pressure test, the concrete specimens with CCCW incorporation exhibited superior impermeability performance [29]. Additionally, it was observed that the second permeability pressure of the impermeable specimens was lower than the first permeability pressure. This observation is likely due to the fact that, although concrete possesses a certain degree of self-healing ability, this healing effect is limited. The self-healing capacity of the impermeable specimens themselves is restricted and unable to restore the original permeability pressure after water pressure damage. Furthermore, the self-healing process of CCCW requires the participation of unhydrated cement particles [31,44]. However, in the later stages of hydration, the amount of unhydrated cement particles decreases, limiting the self-healing capability of the specimens under hydrostatic damage. Generally, the use of CCCW enhances the permeability pressure of the impermeable specimens, particularly with the first permeability pressure of the S1-FC1 specimen reaching up to 1.8 Mpa. This is because the incorporation of CCCW in the mixing process ensures its presence within the concrete from the moment of casting. As a result, CCCW can more effectively react within the concrete, leading to an improvement in the impermeability under pressure. The findings in reference [45] indicate that the incorporation of self-healing materials can improve the impermeability of concrete by 10–21%, which is similar to the results in this study.

**Figure 6:**
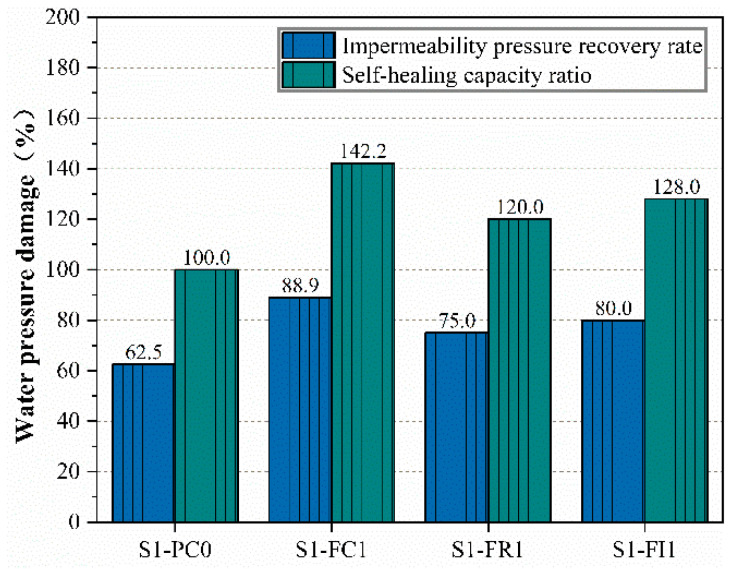
Impermeability pressure recovery rate and self-healing capacity ratio.

To further analyze the effect of CCCW on the self-healing performance of concrete under water pressure damage, Figure 6 shows the permeability pressure recovery rate and water pressure damage self-healing capability ratio of the impermeable specimens. As illustrated, the lowest permeability pressure recovery rate of the specimens with CCCW was still higher than that of the reference concrete. This is because the CCCW incorporated into the concrete reacts to form self-healing products, which fill the voids, increase the penetration pressure, and enhance the concrete’s self-healing capability under water pressure damage [45]. The higher the permeability pressure recovery rate, the greater the water pressure damage self-healing capability ratio, indicating stronger self-healing ability.

The permeability pressure recovery rate of the S1-FC1 specimen is higher than that of the other three groups, at 88.9%. It is evident that the water pressure damage self-healing capability ratio of the S1-FC1 specimen used for curing was the highest, reaching up to 142.2%. This could be due to the spraying of both the water-facing and non-water-facing surfaces of the impermeable specimens after demolding, serving a curing function. Early on, the concrete had numerous voids due to insufficient hydration, allowing the CCCW to penetrate more easily, fill the voids, and significantly increase the permeability pressure and self-healing performance of the concrete [13]. In contrast, the S1-FR1 specimens, which were sprayed after damage for repair, showed lower effectiveness due to the more complete hydration and reduced reactive materials [46], resulting in a lower permeability pressure recovery rate of only 75%. Comparing Figure 5 and Figure 6, it can be seen that the internal mixing type S1-FI1 specimen exhibited higher permeability pressure and self-healing capability than the S1-FR1 specimen, with an 80% permeability pressure recovery rate. This suggests that incorporating CCCW during concrete mixing leads to a more uniform distribution, higher initial permeability pressure, and increased self-healing capability under water pressure damage due to the chemical reactions of active substances in the CCCW [45].

### 3.2. Freeze–Thaw Experiments

**Figure 7:**
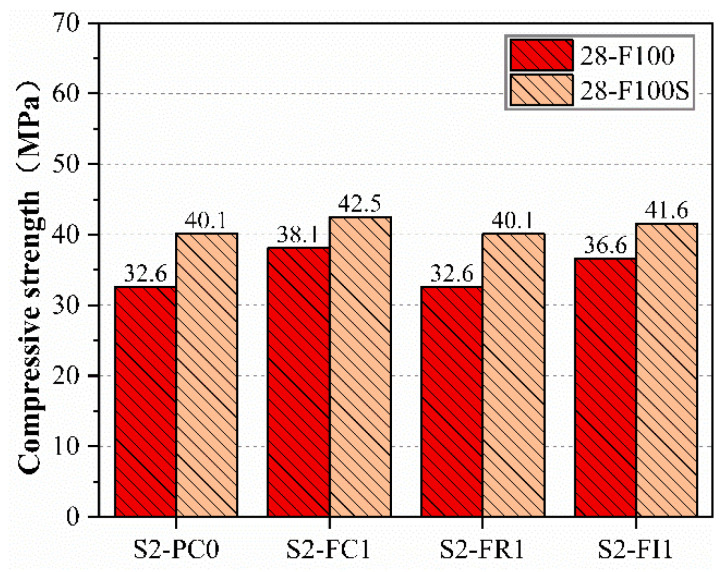
The compressive strength of specimens after 100 freeze–thaw cycles compared to that of specimens of the same age.

After 100 freeze–thaw cycles, the compressive strengths of the freeze–thaw specimens (28-F100) and the corresponding age specimens (28-F100S) are shown in Figure 7. The results indicate that the compressive strength of the freeze–thaw specimens was lower than that of the corresponding age specimens [47] due to the strength reduction caused by freeze–thaw damage. However, compared to the freeze–thaw reference specimens, those with CCCW had higher compressive strengths. For instance, the compressive strength of the S2-FC1 specimen after freeze–thaw cycles remained at 38.1 Mpa, while the S2-PC0 specimen had only 32.6 Mpa. This observation can be attributed to the spraying of S2-FC1 on all surfaces of the freeze–thaw specimens, which acted as a curing agent, made the surface denser, and prevented significant moisture ingress during freeze–thaw cycles, thus maintaining higher compressive strength [48]. The internal mixing type S2-FI1 specimen showed a slightly reduced repair effect on the surface due to reduced repair materials compared to S2-FC1, allowing more water ingress [30]. However, the incorporation of CCCW still reduced internal voids, maintaining a high compressive strength of 36.6 Mpa.

**Figure 8:**
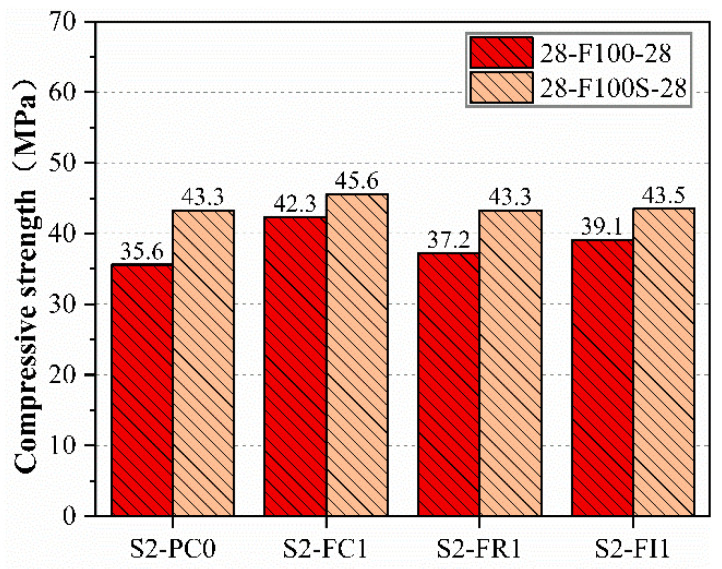
Compressive strength of specimens after 28 days of curing following freeze–thaw cycles compared to that of specimens of the same age.

After curing the freeze–thaw specimens for 28 days, the compressive strengths were tested, as shown in Figure 8. The compressive strengths increased after 28 days of curing, there was a general increase in the compressive strength of the specimens. Among them, the compressive strength of specimens cured for 28 days after freeze–thaw cycles follows a similar pattern to that post-freeze–thaw, where the specimens infused with CCCW exhibit higher compressive strengths than those of S2-PC0. This could be attributed to the fact that the freeze–thaw damage in S2-FC1 specimens was primarily superficial, with minimal internal damage. The compressive strength recovered to 42.3 Mpa for the internally infused S2-FI1 specimens, possibly due to the freeze–thaw cycles allowing water to penetrate the interior and react with the active substances in CCCW, thereby repairing the damage. The damage-repair type S2-FR1 specimens also showed some improvement in repair effectiveness, but, since the application was post-damage, the amount of hydrating substances involved in the reaction was limited, leading to relatively poorer repair outcomes, yet still exceeding the 35.6 Mpa of the control specimen S2-PC0. After a 28-day curing period, the control specimens retained some self-healing capability, but their capacity for repair was limited, resulting in a lower recovery of compressive strength.

**Figure 9:**
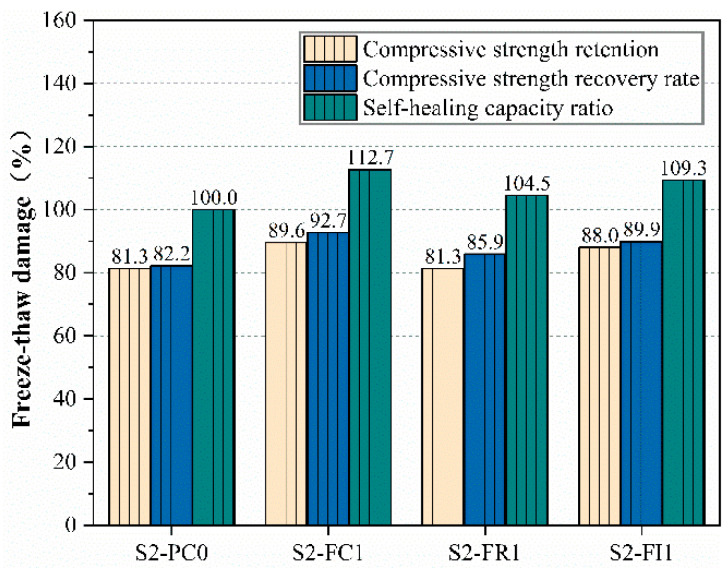
Compressive strength retention, recovery rate, and self-healing capacity ratio.

To further quantitatively assess the influence of incorporating CCCW on the self-healing performance of concrete after freeze–thaw damage, Figure 9 illustrates parameters such as the self-healing capacity ratios for various groups of specimens after freeze–thaw damage.

The results showed that the compressive strength retention rate of the reference specimen S2-PC0 was the lowest at approximately 81.3%, while the curing type S2-FC1 specimen had a retention rate of 89.6%, and the internal mixing type S2-FI1 had 88%, corresponding to the compressive strength results after freeze–thaw cycles.

After 28 days of curing, the freeze–thaw specimens showed an increase in compressive strength, with the CCCW-incorporated specimens exhibiting higher recovery rates than the reference specimens. The highest freeze–thaw damage self-healing capability ratios were observed in the S2-FC1 and S2-FI1 specimens, indicating superior self-healing performance, while the S2-FR1 specimen showed a relative improvement, but still lower than the other CCCW specimens. This indicates that using the curing-type CCCW mixing method for concrete exposed to freeze–thaw damage results in the best self-healing performance. The findings in [49] are consistent with those in this study. Reference [49] demonstrated that, after concrete undergoes freeze–thaw damage, surface spalling occurs. However, when CCCW is applied to the surface, the freeze–thaw resistance significantly improves. This is because the application of CCCW on the surface effectively reduces the ingress of moisture into the concrete, thereby mitigating the occurrence of freeze–thaw damage, while also facilitating the repair of the already damaged surface areas.

### 3.3. Pre-Pressure Experiments

**Figure 10:**
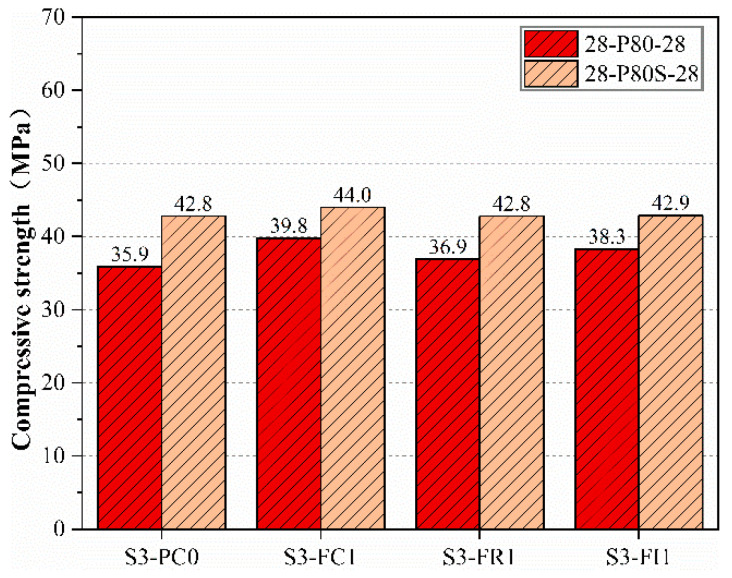
Compressive strength of specimens after 28 days of curing following pre-pressure compared to that of specimens of the same age.

The specimens for this test were prepared by the procedure mentioned in Section 2.5. The compressive strength of the load-damaged specimens after 28 days of curing (28-P80-28) and the corresponding age control specimens (28-P80S-28) are shown in Figure 10. As the curing time increased, the compressive strength of the corresponding age specimens did not vary significantly, indicating that CCCW had minimal effect on the late-stage compressive strength. However, the compressive strength of the load-damaged specimens with CCCW was significantly higher than that of the reference specimens, especially the S3-FC1 specimen, which reached 39.8 Mpa, demonstrating better performance in compressive strength recovery after load damage. Applying 80% of the compressive strength load induced microcracks in both the interior and surface, and both internal mixing and curing CCCW could repair these cracks, reacting with water to fill voids and densify the structure, thereby improving the compressive strength of the load-damaged specimens after curing.

The S3-FR1 specimen showed slightly higher compressive strength after 28 days of curing compared to the reference specimen S3-PC0. This may be because the S3-FR1 specimen was sprayed after load damage, with fewer reactive materials penetrating deep into the specimen due to extensive internal damage and limited reaction time of only 28 days. However, it still showed some repair effect, being 1 Mpa higher than the reference specimen. Meanwhile, the S3-FC1 and S3-FI1 specimens showed significant recovery in compressive strength after 28 days of standard curing, indicating that CCCW incorporation enhances the compressive strength of concrete specimens after load damage.

**Figure 11:**
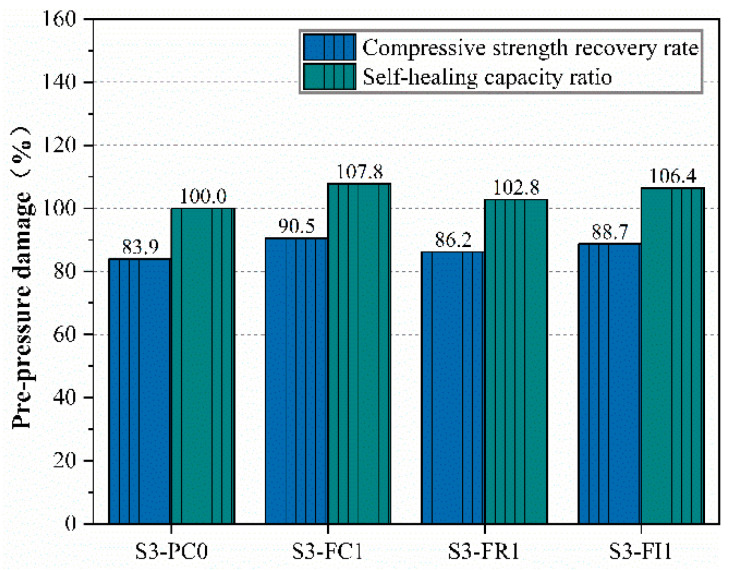
Compressive strength recovery rate and self-healing capacity ratio.

Figure 11 shows the load damage compressive strength recovery rate and self-healing capability ratio. The results indicate that even the lowest load damage compressive strength recovery rate of the CCCW-incorporated specimens (86.2%) was higher than that of the reference specimen (83.9%). The S3-FC1 specimen reached 90.5%, while the internal mixing type S3-FI1 achieved 88.7%. The higher the load damage compressive strength recovery rate, the greater the self-healing capability ratio and self-healing ability. In this experiment, the partial replacement of water in the S3-FI1 specimen reduced the initial water–cement ratio, resulting in higher compressive strength. After load damage, the active substances in the CCCW reacted to restore compressive strength. The self-healing performance of the S3-FR1 specimen was slightly higher than that of the reference specimen S3-PC0, indicating that post-damage spraying has some repair effect but is not as effective as spraying after demolding, as seen in the S3-FC1 specimen. Overall, CCCW incorporation improved the compressive strength of concrete specimens after load damage and enhanced the self-healing capability ratio. On the other hand, both the curing-type CCCW mixing method and the internally incorporated CCCW mixing method show good performance in healing load-induced damage. This is because the reactive chemical components in CCCW react with calcium ions in the damaged concrete, producing healing products that fill the cracks at the damaged areas, thus inducing the self-healing effect [21]. Therefore, the existing literature has shown that incorporating CCCW into concrete can effectively repair surface damage in tunnel lining concrete structures [50], which is consistent with the findings in this study.

### 3.4. Crack Self-Healing Evaluation

#### 3.4.1. Water Permeability of the Specimens Healed in Standard and Immersion Curing

**Figure 12:**
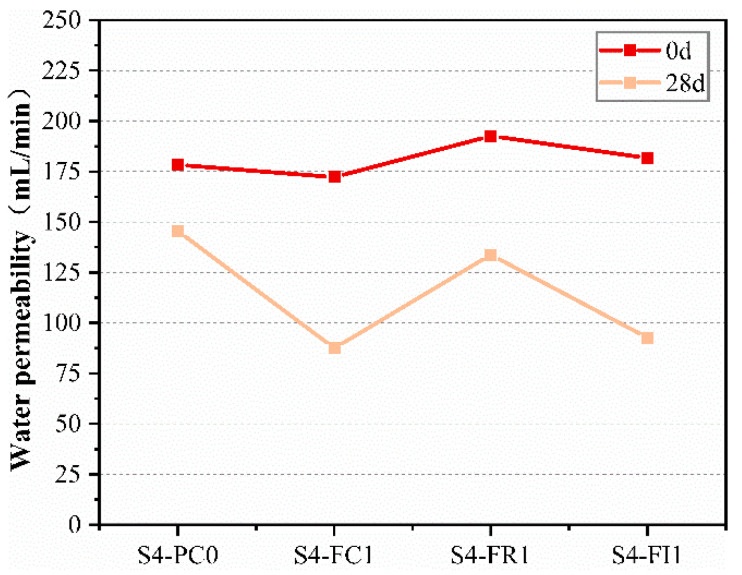
Water permeability measured at 0 d and 28 d of standard curing.

To further evaluate the effect of CCCW on crack self-healing performance, the water permeability was used as an indicator. Figure 12 shows the permeability of specimens with cracks under standard curing. Before healing, the permeability of each specimen was similar due to the requirements for water permeability. Microscopic observation of the crack mouth showed that deeper crack areas might not be visible under the microscope, affecting the changes in permeability. As shown in Figure 12, the permeability of each specimen decreased with curing time, indicating a certain self-healing ability. After 28 days of curing, significant changes in permeability were observed. Except for the reference group S4-PC0 and the curing type S4-FR1 specimens, the permeability of the other CCCW-incorporated specimens decreased significantly compared to before healing, especially the curing type S4-FC1 specimen, which had a final permeability of only 87.5 mL/min. This may be due to the spraying of CCCW on the surface penetrating the specimens and filling the cracks. However, the S4-FR1 specimen also had high initial permeability, resulting in a higher final permeability. This suggests that the cracks were not fully healed after curing, but CCCW still participated in the reaction under water, repairing the cracks and reducing the final water permeability [51], thus improving permeability.

**Figure 13:**
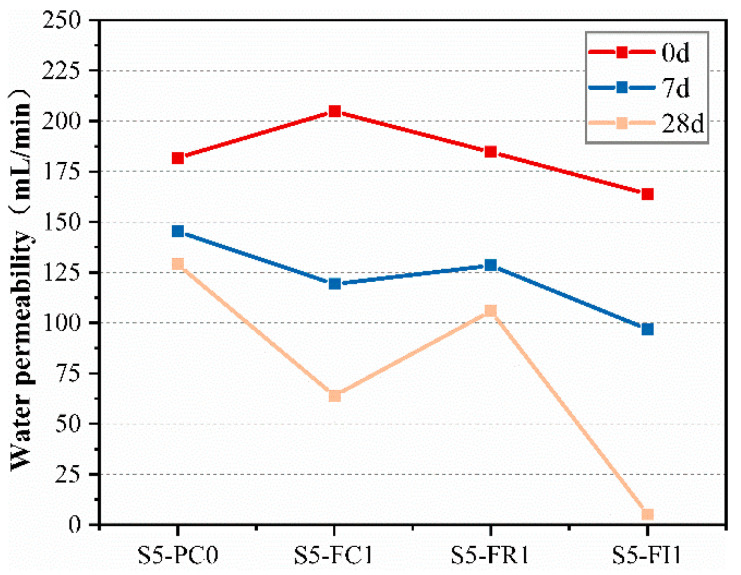
Water permeability measured at 0 d, 7 d, and 28 d of water-immersion curing.

To compare the crack self-healing performance of different CCCW incorporation methods under different curing conditions, water-immersion curing was employed, and the water permeability of various pre-cracked specimens were tested at 0, 7, and 28 days, as shown in Figure 13. The results showed a similar pattern to standard curing, with decreasing seepage rates over time. The final water permeability was lower under water-immersion curing, indicating better self-healing performance. After 28 days of water-immersion curing, the final permeability of the curing type S5-FC1 specimen was 63.9 mL/min, lower than under standard curing, indicating enhanced crack self-healing performance under adequate water conditions. The internal mixing type S5-FI1 specimen had a final permeability of only 52 mL/min, attributed to lower initial water permeability and improved repair effects, with superior self-healing performance compared to S5-FC1.

#### 3.4.2. Healing Ratio

To better explore the effect of CCCW incorporation methods on crack self-healing performance under standard and water-immersion curing, the relative permeability coefficient and crack self-healing capability ratio were analyzed.

**Figure 14:**
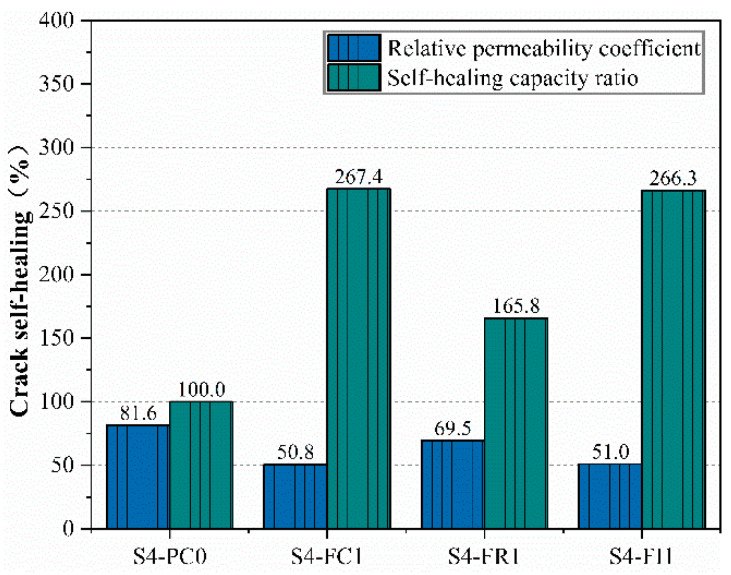
Relative permeability coefficient and self-healing capacity ratio in standard curing.

Figure 14 shows the relative permeability coefficient and self-healing capability ratio of pre-cracked specimens under standard curing. The S4-FC1 specimen had a relative permeability coefficient of only 50.8%, close to S4-FI1 at 51%, indicating a rapid reduction in permeability and low final seepage rates, consistent with the results in Figure 12. The S4-FR1 specimen had a higher relative permeability rate, corresponding to higher final permeability, but still lower than the reference group S4-PC0, indicating that post-damage spraying can repair cracks to some extent.

Furthermore, the relative permeability coefficients of the CCCW-incorporated specimens were significantly lower than the reference specimens, likely due to the exposure of internal cracks in the curing environment, allowing more reactive materials to participate in the reaction and improve repair effects. The S4-FC1 specimen exhibited both surface and internal repair effects, leading to better crack repair. The S4-FI1 specimen showed similar repair effects to the S4-FC1 specimen, demonstrating good crack self-healing performance.

**Figure 15:**
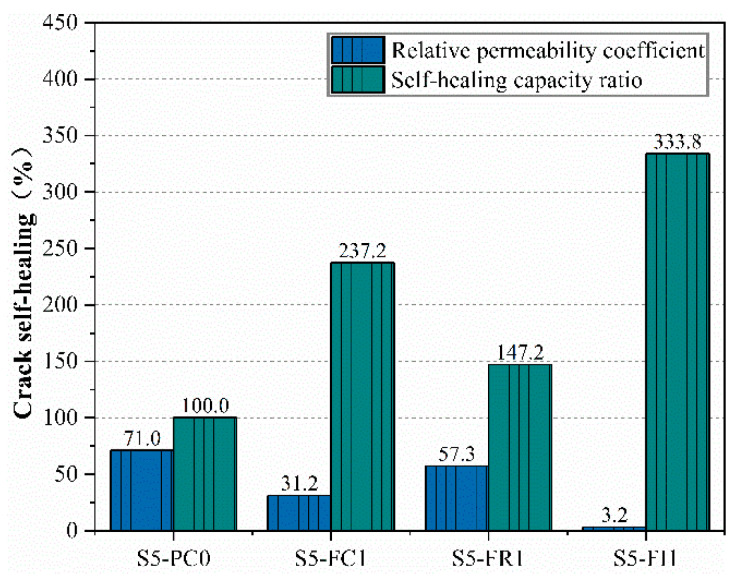
Relative permeability coefficient and self-healing capacity ratio in water-immersion curing.

Comparing the crack self-healing performance of different CCCW incorporation methods under two curing conditions, the results of pre-cracked specimens under water-immersion curing are shown in Figure 15. The S5-FI1 specimen exhibited the best self-healing performance, attributed to the abundant water promoting more reactions at the crack site and the presence of hydration heat inhibitors reducing initial hydration rates. The curing type S5-FC1 specimen also showed a high self-healing capability ratio due to surface spraying after demolding, maintaining a low relative permeability coefficient despite a higher initial water permeability. Comparing the relative permeability coefficients under standard and water-immersion curing, the latter had lower values, indicating better self-healing performance. A similar observation was reported in [21], where concrete specimens incorporating CCCW exhibited the highest crack self-healing rate under water-immersion conditions. Even for larger initial crack widths (approximately 0.25 mm), near-complete healing was achieved.

#### 3.4.3. Surface Crack Width

**Figure 16:**
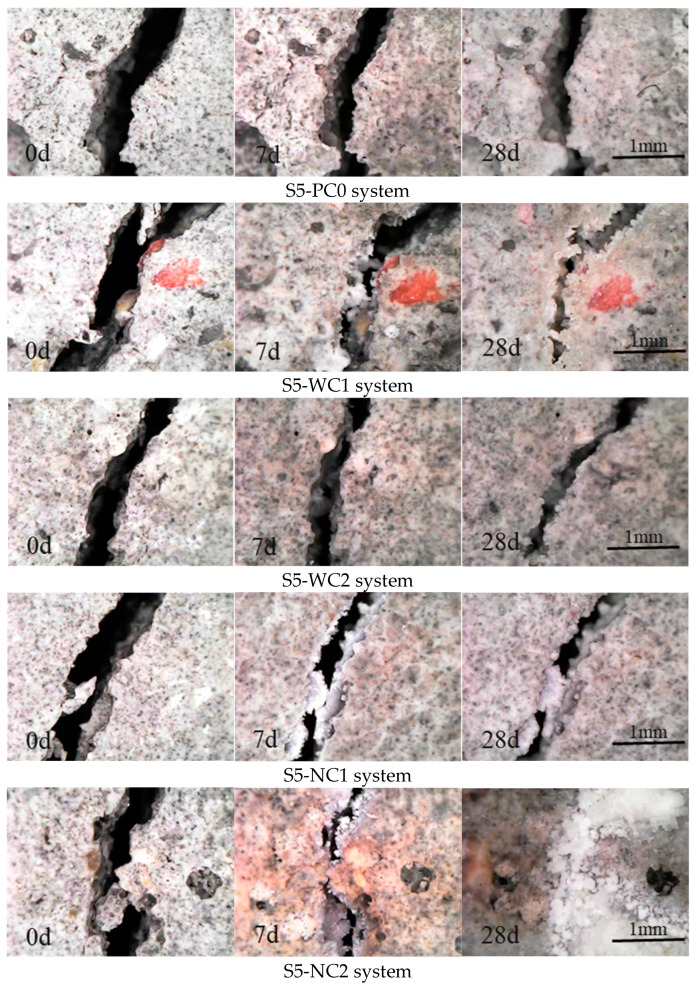
Evolution of visual crack closure after healing in water-immersion curing.

**Figure 17:**
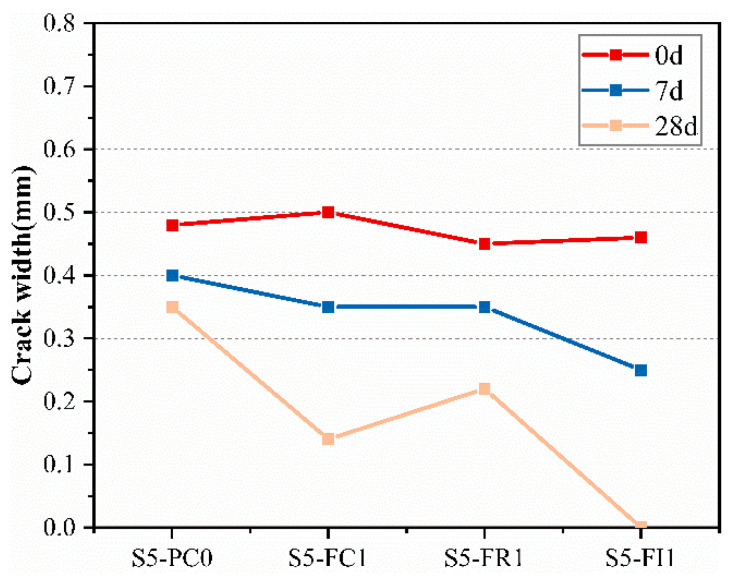
The crack width at different ages of water-immersion healing.

To accelerate the crack self-healing rate, the specimens were cured by water immersion. The self-healing process of the cracks was observed using a WiFi digital microscope produced by Suzhou Dexinshun Trading Co., Ltd., Suzhou, China. The device has a magnification range of 50–1000× and a pixel resolution of 640 × 480. Figure 16 depicts visual images of the cracks at different measurement times under water-immersion curing. The initial crack width at 0 days was measured, and subsequent measurements were taken at 7 and 28 days, as shown in Figure 17. Healing products were observed to grow from the crack sides toward the center, filling the cracks, with some cracks fully closed after a period of curing.

The white healing products, mainly formed within the cracks over 28 days, were observed more abundantly in the CCCW-incorporated specimens than in the reference specimen S5-PC0 [52]. This confirms the significantly lower permeability of CCCW specimens compared to the reference specimen under the same curing period. The S5-FR1 specimen showed minimal healing products, explaining its limited reduction in seepage rate, while other CCCW-incorporated specimens had cracks filled with healing products [53], significantly reducing final permeability. At 7 days, the products appeared to be loose in the cracks, with density increasing over time. The S5-FI1 specimen showed clear crack filling with white healing products, corresponding to its lower final permeability and stronger self-healing performance.

**Figure 18:**
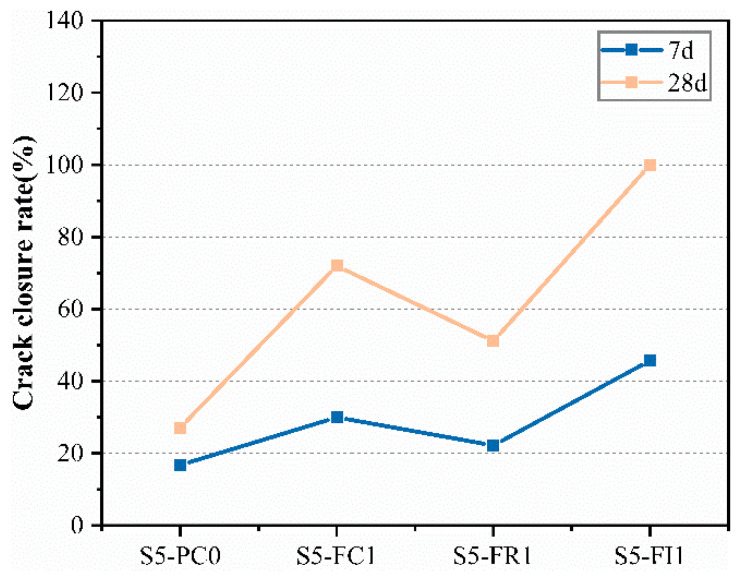
The crack closure at different ages.

Figure 18 shows the crack closure rate (γ) determined by Equation (11). Higher γ values indicate better healing and crack closure. The surface crack width of pre-cracked specimens significantly decreased at 28 days, consistent with the corresponding age permeability results. The CCCW-incorporated specimens showed greater reductions in crack width and higher crack closure rates than the reference specimen S5-PC0. The S5-FC1 and S5-FI1 specimens exhibited the best crack healing, with the S5-FI1 specimen showing nearly complete crack closure after 28 days of water-immersion curing. The reduction in crack width was attributed to the active substances in the CCCW reacting to form healing products, filling the cracks and improving the crack closure rate [41]. According to [54], the incorporation of CCCW into tunnel-lining concrete structures has been shown to effectively promote the hydration of cement. This process leads to the formation of a large number of branched and needle-like crystals within the concrete, which significantly enhances the self-healing of cracks in the lining concrete.

### 3.5. Microscopic Test

#### 3.5.1. Microscopic Morphology and Chemical Composition of the Healing Product

**Figure 19:**
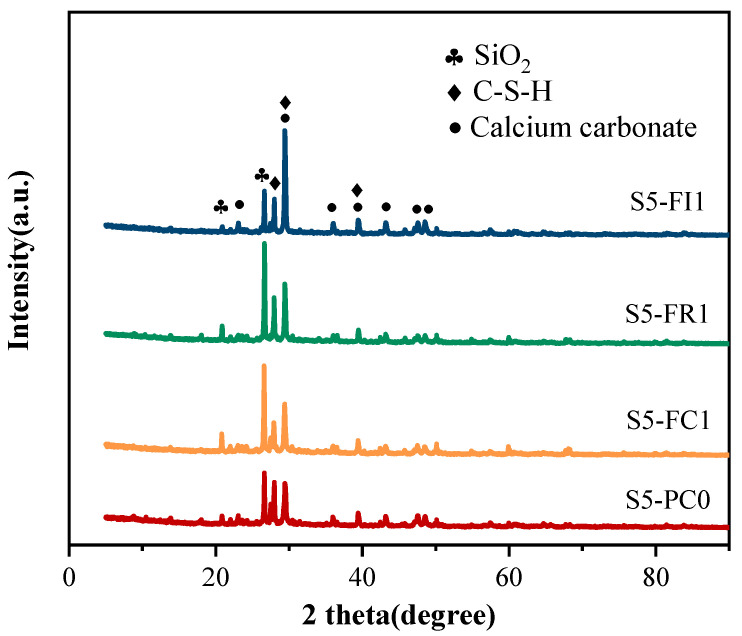
XRD patterns of the healing products of the reference specimen and the specimen incorporated with CCCW.

To determine the main self-healing mechanism of cracks in the CCCW system, the healing products on the crack walls after 28 days of water-immersion curing were ground into powder, and their phase composition was measured by XRD. The diffraction patterns of the healing products from different CCCW specimens are shown in Figure 19. The XRD experimental results are consistent with those reported in [29,55], where the self-healing products within the cracks are primarily composed of CaCO_3_ and C-S-H. A qualitative analysis of the XRD patterns showed the presence of these substances in the healing products. The intensity of the peaks qualitatively reflected the content of the components. Compared to the reference concrete, the CCCW specimens exhibited larger diffraction peaks for healing products, indicating higher concentrations of calcium carbonate and C-S-H. The highest peak was observed in the S5-FI1 specimen, indicating a significant amount of healing products at the crack site, consistent with the macro-test results and the self-healing performance of the S5-FI1 specimen, showing that CCCW promotes the formation of healing products, thereby improving crack self-healing performance.

**Figure 20:**
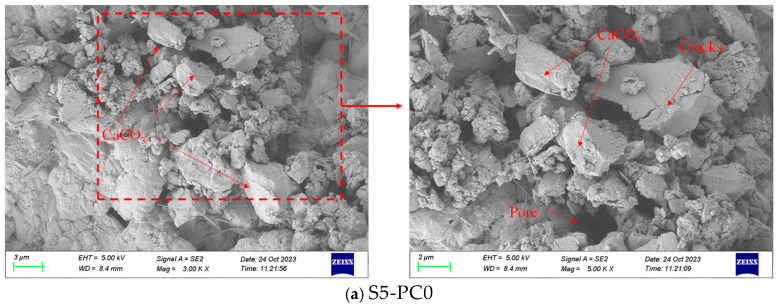

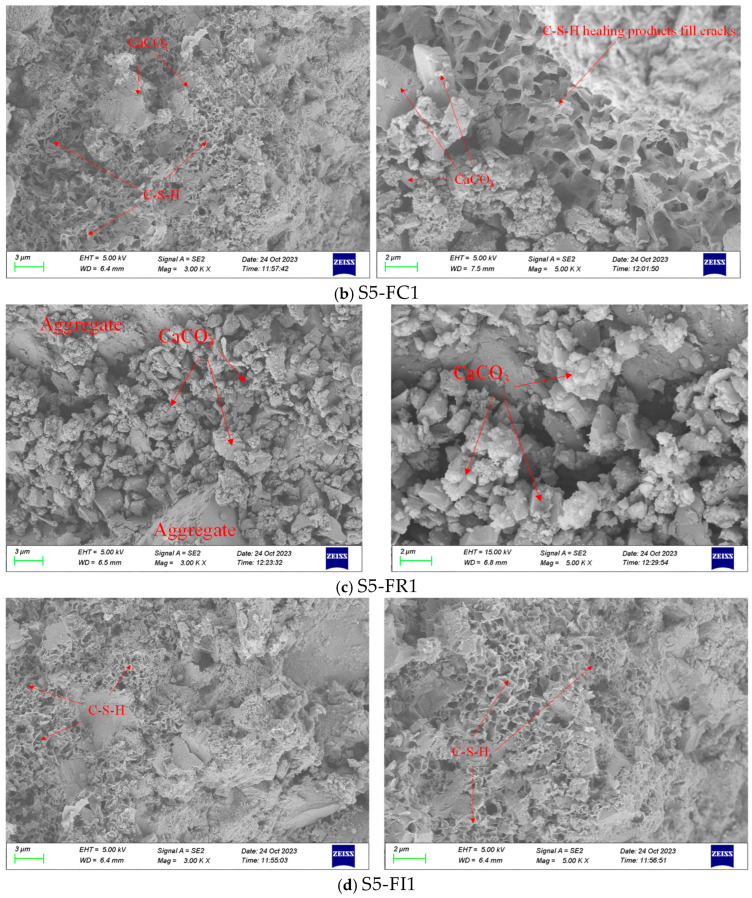
SEM images of the interior of the cracks.

To further understand the healing mechanism, SEM was used to observe the morphology of the healing products on the crack surfaces. Figure 20 shows the morphology of healing products in cracks from different CCCW specimens. Based on the XRD test results and the microstructural characteristics of self-healing products from the literature [29], the corresponding components of the self-healing products in this study are highlighted in Figure 20. The microstructures of the CCCW specimens were denser, with smaller particles and a significantly increased amount of healing products, indicating that CCCW can improve the microstructure of concrete crack-healing products. The healing products in the S5-PC0 and S5-FR1 systems mainly consisted of irregular particles of different sizes, with larger and more loosely packed particles in the S5-PC0 and more densely packed particles in the S5-FR1. The S5-FC1 and S5-FI1 systems also showed some particles, but with more flocculent shapes and denser structures, indicating better healing effects under water-immersion curing, especially in the S5-FI1 specimen, where the crack surfaces were filled with healing products, showing superior crack self-healing performance.

#### 3.5.2. Analysis of the Self-Healing Reaction Process

The self-healing products originate from the further hydration of unhydrated cement particles and reactions with carbon dioxide in the air, forming new C-S-H gel and calcium carbonate to fill the cracks. Based on the sources of self-healing products, Table 3 lists the reaction equations involved in the self-healing process.

**Figure 21:**
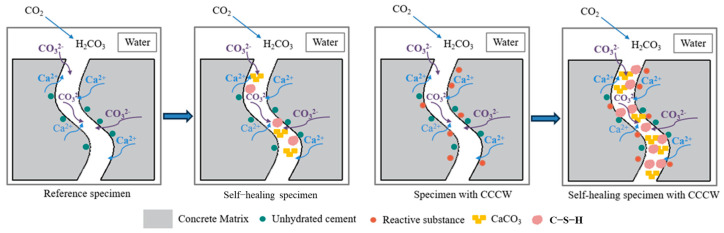
Schematic illustration of self-healing mechanism of cracks.

Based on the test results in this study, the mechanisms of crack healing were analyzed, as shown in Figure 21. When the reference specimen cracks were prefabricated, the chemical reactions listed in Table 2 occurred under water-immersion curing, generating calcium carbonate and C-S-H gel. However, the limited amount of unhydrated cement particles resulted in suboptimal crack healing, consistent with the images of the healing process in Figure 16. With CCCW, additional reactions occurred beyond those in the reference specimen, as shown in Figure 21. The active substances in CCCW reacted with calcium ions in the concrete, forming a complex. When encountering silicate ions, new healing products were generated. Moreover, the abundant water accelerated the reaction, significantly increasing the amount of healing products compared to the reference specimen. During the healing reaction, the continuous complexation of calcium ions increased the calcium ion content at the crack site, further promoting the formation of healing products. This explains the denser microstructure of the CCCW specimens and the higher amount of healing products in the cracks, consistent with the observed self-healing performance.

## 4. Conclusions

This study investigates the effects of different CCCW incorporation methods on the self-healing performance of concrete under various damage conditions. Based on the experimental results, the following conclusions can be drawn:

1. Concrete specimens incorporating CCCW exhibited higher first and second permeability pressures compared to the control specimens. These specimens also demonstrated higher permeability pressure recovery rates and superior water pressure damage self-healing capabilities, indicating enhanced impermeability. Among the specimens, the S1-FC1 specimen exhibited the highest water pressure resistance;
2. After 100 freeze–thaw cycles, the CCCW-incorporated specimens showed higher compressive strengths than the reference specimens, suggesting that CCCW effectively mitigated moisture ingress and enhanced freeze–thaw resistance. After 28 days of curing, the CCCW specimens exhibited superior compressive strength recovery rates, with the S2-FC1 specimen achieving 92.7%, highlighting its excellent self-healing performance under freeze–thaw damage;
3. In the load damage tests, there was no significant difference in the compressive strength of specimens at 28 days across different mixing methods, indicating that the effect of CCCW on late-stage strength improvement was minimal. However, specimens with CCCW incorporation demonstrated a higher recovery rate of compressive strength post-load damage, with the S3-FC1 and S3-FI1 specimens recovering 90.5% and 88.7%, respectively;
4. Both standard curing and water-immersion curing showed a reduction in seepage rates over time, with water-immersion curing resulting in a greater decrease. This suggests that water-immersion curing provided better self-healing performance. The reference concrete exhibited lower crack closure rates, significantly less than those in CCCW-incorporated specimens. Notably, the S5-FI1 specimen displayed considerable healing products and achieved notable crack self-healing;
5. Crack healing requires the formation of stable calcium carbonate and C-S-H gel crystalline products. These products form a dense structure that promotes better crack healing due to their tightly packed morphology. The presence of a substantial amount of flocculent morphology further supports the self-healing of cracks;
6. For different damage types in hydraulic engineering, the curing-type FC1 and internal mixing-type FI1 are preferred for water pressure, freeze–thaw, and load damage applications. The internal mixing-type FI1 is particularly suitable for structures prone to frequent cracking, while the FR1 type can be used for post-damage spraying repairs.

## Figures and Tables

**Table 1:** List of symbols.

Symbol	Definition
CCCW	Cementitious Capillary Crystalline Waterproofing Materials
SEM	Scanning electron microscopy
XRD	X-ray Diffraction
CaCO_3_	Calcium carbonate
C-S-H	Calcium silicate hydrate
PC0	Reference concrete
FI1	The concrete formed with internally mixed CCCW
FC1	The concrete formed with CCCW spray curing after demolding
FR1	The concrete repaired with spray-applied CCCW after damage.
S1	Water pressure damage
S2	Freeze–thaw damage
S3	Load damage
S4	Cracking damage
S5	Crack self-healing test
$P_{1}$	The first permeability pressures
$P_{2}$	The second permeability pressures
$\lambda_{w}$	The permeability pressure recovery rate
$\lambda_{ws}$	The permeability pressure recovery rates of concrete mixed with CCCW
$\lambda_{wc}$	The permeability pressure recovery rates of reference concrete
$R_{w}$	The water pressure damage self-healing capability ratio
$f_{c}$	The compressive strength of specimens after 100 freeze–thaw cycles
$f_{i}$	The compressive strength of control specimens at the same age
$f_{n}$	The compressive strength of specimens after 100 freeze–thaw cycles and 28 days of curing
$f_{0}$	The compressive strength of control specimens at the same age as the freeze–thaw cured specimens
η	The freeze–thaw damage compressive strength retention rate
$\lambda_{f}$	The freeze–thaw damage compressive strength recovery rate
$\lambda_{fs}$	The freeze–thaw damage compressive strength recovery rates of concrete mixed with CCCW
$\lambda_{fc}$	The freeze–thaw damage compressive strength recovery rates of reference concrete
$R_{f}$	The freeze–thaw damage self-healing capability ratio
$f_{l}$	The compressive strength of load-damaged specimens after 28 days of curing
$f_{l0}$	The compressive strength of control specimens at the same age
$\lambda_{p}$	The load damage compressive strength recovery rate
$\lambda_{ps}$	The load damage compressive strength recovery rates of concrete mixed with CCCW
$\lambda_{pc}$	The load damage compressive strength recovery rates of reference concrete
$R_{p}$	The load damage self-healing capability ratio
K	Seepage rate
$K_{n}$	The final seepage rates
$K_{0}$	The initial seepage rates
V	The remaining water volume in the rubber cylinder
t	The test duration
β	The relative permeability coefficient
$\beta_{s}$	The relative permeability coefficients of concrete mixed with CCCW
$\beta_{c}$	The relative permeability coefficients of reference concrete
$R_{c}$	The crack self-healing capability ratio
$W_{0}$	The initial width of the pre-crack
$W_{n}$	The crack width after *n* days of self-healing
γ	The crack closure rate

**Table 2:** Mix proportions of the concrete.

Mix ID	Cement (kg/m^3^)	Water (kg/m^3^)	Sand (kg/m^3^)	Aggregate (kg/m^3^)	Water Reducing Agent (kg/m^3^)	CCCW Mixing Method
PC0	355	195	629	1221	1.34	0
FI1	355	187.9	629	1221	1.34	Internal mixing (2%)
FC1	355	195	629	1221	1.34	Curing (250 mL/m^2^)
FR1	355	195	629	1221	1.34	Repair (250 mL/m^2^)

**Table 3:** Self-healing reactions.

Source of Self-Healing Products	Self-Healing Reaction
Rehydration of unhydrated cement [55]	2C3S + 6H → C-S-H + 3CH 2C3S + 4H → C-S-H + CH
Precipitation of calcium carbonate [30]	CH + CO_2_ → CaCO_3_

Notes: C = CaO, S = SiO_2_, H = H_2_O, CH = Ca(OH)_2_.

## Data Availability

All data has been shown in this manuscript. Further inquiries can be directed to the corresponding author.
